# Assessing intersectional gender analysis in Nepal’s health management information system: a case study on tuberculosis for inclusive health systems

**DOI:** 10.1186/s40249-024-01194-4

**Published:** 2024-04-25

**Authors:** Ayuska Parajuli, Sampurna Kakchapati, Abriti Arjyal, Deepak Joshi, Chandani Kharel, Mariam Otmani del Barrio, Sushil C Baral

**Affiliations:** 1 HERD International, Saibu Awas Cr-10 Marga, Bhaisepati, Lalitpur, Nepal; 2 UNICEF/UNDP/World Bank/WHO Special Programme for Research and Training in Tropical Diseases (TDR), World Health Organization, Geneva, Switzerland

**Keywords:** Tuberculosis, Intersectional gender analysis, Gender and social inequities, Social determinant, Health Management Information System, National Tuberculosis Programme, Social inclusion, Gender

## Abstract

**Background:**

Tuberculosis (TB) remains a major public health problem in Nepal, high in settings marked by prevalent gender and social inequities. Various social stratifiers intersect, either privileging or oppressing individuals based on their characteristics and contexts, thereby increasing risks, vulnerabilities and marganilisation associated with TB. This study aimed to assess the inclusiveness of gender and other social stratifiers in key health related national policies and the Health Management Information System (HMIS) of National Tuberculosis Programme (NTP) by conducting an intersectional analysis of TB cases recorded via HMIS.

**Methods:**

A desk review of key policies and the NTP’s HMIS was conducted. Retrospective intersectional analysis utilized two secondary data sources: annual NTP report (2017–2021) and records of 628 TB cases via HMIS 6.5 from two TB centres (2017/18–2018/19). Chi-square test and multi-variate analysis was used to assess the association between social stratifers and types of TB, registration category and treatment outcome.

**Results:**

Gender, social inclusion and concept of intersectionality are incorporated into various health policies and strategies but lack effective implementation. NTP has initiated the collection of age, sex, ethnicity and location data since 2014/15 through the HMIS. However, only age and sex disaggregated data are routinely reported, leaving recorded social stratifiers of TB patients static without analysis and dissemination. Furthermore, findings from the intersectional analysis using TB secondary data, showed that male more than 25 years exhibited higher odds [adjusted odds ratio (a*OR*) = 4.95, 95% confidence interval (*CI*): 1.60–19.06, *P* = 0.01)] of successful outcome compared to male TB patients less than 25 years. Similarly, sex was significantly associated with types of TB (*P* < 0.05) whereas both age (*P* < 0.05) and sex (*P* < 0.05) were significantly associated with patient registration category (old/new cases).

**Conclusions:**

The results highlight inadequacy in the availability of social stratifiers in the routine HMIS. This limitation hampers the NTP’s ability to conduct intersectional analyses, crucial for unveiling the roles of other social determinants of TB. Such limitation underscores the need for more disaggregated data in routine NTP to better inform policies and plans contributing to the development of a more responsive and equitable TB programme and effectively addressing disparities.

## Background

Nepal, in its early stage of federalisation, is a multi-ethnic, multi-lingual, multi-religious and multi-cultural state with diverse geography. The new state architecture comprises three tiers of government—one federal, seven provincial and 753 local governments. In this federal structure, health is among the most decentralized sectors, where basic health services fall under the exclusive functions of local government [1, 2]. The local governments have the authority to plan, operate, and manage their own health systems, bringing health services closer to peoples’ home. This approach aims to narrow gaps in health service access and utilization caused by synergistic interaction with various social stratifiers, such as gender, education, occupation and socio-economic status of the individual [3, 4].

Intersectional gender analysis involves analyzing how gender power relations intersect with other social factors (such as age, ethnicity, religion, gender, education, occupation, geography, migration status, etc.) to affect people’s lives and create differences in needs and experiences [5]. These factors intersect, either privileging or oppressing individuals based on their characteristics and contexts, thereby increasing the risk, vulnerabilities, and marginalisation. Such evidence can better inform policies, programmes and services to ensure that no one is left behind. Tuberculosis (TB) servs as an example, as National TB compared against yearly disease estimation by WHO shows that 10,000 TB patients are beyond the reach of the National Tuberculosis Programme (NTP), Nepal [6].

TB remains a public health challenge in Nepal. As of 2021, Nepal is one of the high TB burden countries, with an increasing prevalence of cases. A total of 28,677 cases were notified and registered within the NTP in 2020/21 [7]. National data indicates that males suffer two times more from TB than females [7]. The higher prevalence of TB among males is attributed to sex and gender specific behavioral factors, such as daily activities/occupation, risk behaviors, social roles and responsibilities [8, 9]. Males travel more frequently, leading to more social contacts; spend more time in settings conducive for TB transmission (e.g. bars) and engaged in occupations associated with a higher risk of infection, such as mining, labor work [8, 9].

TB is high settings with common practice of gender and social inequities. Lower TB prevalence among females may suggest under reporting and underdiagnosis [10, 11]. Women in Nepal experience a longer total delay before TB diagnosis (median 3.3 months) compared to men (2.3 months) [12]. Limited household decision‐making power to females, particularly regarding healthcare, may contribute to this delay. In 2016, over 40% of women could not make decisions about their own healthcare due to reasons such as treatment cost, distance to health facilities, and lack of permission to seek treatment [13]. Apart from gender disparities, patients from rural areas experience longer delays in seeking care compared to the urban population [14]. Social barriers to the healthcare access include fear of stigma and discrimination, linked to poverty, lower caste and TB [15]. Moreover, treatment outcomes of TB are also associated with sex, gender, age, education, race/ethnicity and residential area of TB patients [16]. Although TB drugs are provided free of cost, several disabling factors such as poor socioeconomic conditions, family liabilities, and the burden of losing income contribute to loss to follow up during TB treatment [17].

Taking TB as a case example, these literature findings provide evidence that various social stratifiers such as age, sex, education, occupation, gendered roles and responsibilities, largely influence the disease and treatment outcomes [7–17]. Additionally, traditionally inherited caste-based discrimination is one of the major barriers to accessing healthcare services in Nepal, often interacting synergistically with gender, education, occupation and socio-economic status of individuals [3, 4]. Therefore, the application of an intersectional gender lens is critical to improving the health status of people, including those with TB. A toolkit for conducting intersectional gender analysis for infectious diseases of poverty has been established to comprehend concerns via an intersectional gender lens. This pilot study aimed to inform the toolkit "Incorporating Intersectional Gender Analysis into Research on Poverty-Related Infectious Diseases" [27]. The study objective was to explore inclusiveness of gender and social stratifiers in key health-related policy documents and Health Management Information System (HMIS) through a desk review. Using TB as a case example, this study also aimed to assess the feasibility of conducting disaggregated and intersectional analysis of TB patient data recorded via HMIS within the NTP in Nepal.

## Method

### Study design

A retrospective study was conducted through desk review and secondary data analysis. A web-based search was performed on key health-related policy documents of relevant government ministries. The reviewed policy documents included the Constitution of Nepal (2015), Urban Health Policy (2015), National Population Policy (2014), National Health Policy (2019), National Strategy for Reaching the Unreached (2016), Gender Equality and Social Inclusion Strategy of the Health Sector (2018), and Health Sector Information System National Strategy (2006). The review was focused to explore inclusiveness of gender, equity and social stratifiers within the policies and strategies [18–24]. Additionally, National strategy related to TB, namely National Strategic Plan for Tuberculosis Prevention, Care and Control 2016–2021 and the National Strategic Plan to End TB 2021/22–2025/26 was reviewed, considering TB as a case example in this study [6, 25].

Moreover, HMIS was reviewed to explore the availability of various social stratifiers, with a specific focus on TB as a case example [26]. TB data reported over the last five years (2017–2021) were obtained from the website of National Tuberculosis Control Center (NTCC) for age-and -sex disaggregated trend analysis. Similarly, secondary analysis of recorded TB patients from two fiscal years (2017/18–2018/19) from two Directly Observed Therapy, Short-course (DOTS) centers was carried out to explore how sex, age and ethnicity interact with each other, shaping the treatment of TB patients enrolled in the NTP.

### Study setting

Two selected DOTS centers were identified in the Metropolitan City of Kathmandu district in Bagmati province. This province had the highest number of notified TB patients in the year 2020/21 (6664) compared to other provinces in Nepal [7]. Among the districts within this province, Kathmandu alone accounted for around 44% (2982 TB cases), contributing approximately 10.39% to the national total (28,677) [7].

According to the 2021 National Housing and Population Census, Kathmandu district had a total population of 2,017,532, with the male population (1,025,727), slightly outnumbering the female population (991,805) [27]. This district experiences high migration from various parts of the country. There were 45 DOTS center in Kathmandu, located in various hospitals, referral centers and Urban Health Clinics (UHC) [28, 29]. Based on the information obtained for the fiscal year 2017/18 and 2018/19, Swayambhu UHC was one of the UHCs with a high case load, handling approximately 200–250 TB cases per year. Similarly, a referral center named Nepal Anti-Tuberculosis Association (NATA), supported by the German Nepal Tuberculosis Project (GENETUP), was the largest TB referral center in Kathmandu, linked to various DOTS centres. This referral center documented approximately 400–450 cases in the last two fiscal years- 2017/18, 2018/2019. Therefore, considering feasibility with limited time and resources, we purposively selected Swayambhu UHC and NATA for this study based on their TB case load.

### Data collection

All DOTS centers follow HMIS 6.5 as the TB treatment register. The same template was created in an excel sheet for data collection. Before data collection, coordination meetings with officials of the NTCC and the Epidemiology and Disease Control Division (EDCD) of the Department of Health Services, Ministry of Health and Population were conducted. During these meetings, stakeholders were oriented about the study objectives and methodology. Official letters of support were received from the respective government offices, facilitating communication with officials at DOTS centres. Similarly, clinic supervisors and DOTS focal persons from the study sites were oriented on the objectives and methodology of the study.

Supervisors, who were the data custodians at the DOTS centers, were requested to provide anonymised data during data collection. Each patient was assigned a unique identification number and no other patient identifiable information was obtained during the data collection. Data was collected and entered at the DOTS center in the presence of data custodian. Any missing information in the register was immediately discussed with the data custodian, who validated data with other records of the respective patient present in the DOTS center. Data collection took place over a period of one month in July 2020.

### Data management and analysis

MS Excel was used for the data cleaning, while statistical software STATA version 14 (StataCorp LLC, College Station, Texas, USA), and the R Programme (Lucent Technologies, Jasmine Mountain, USA) were used to analyse data and create graphs, respectively. The study variables were patients’ age, sex, ethnicity, types of TB, patient registration category (new or old cases), and treatment outcome. Ethnicity of patient was categorised into two groups: ‘advantaged caste groups’ and ‘dis-advantaged caste groups’ [30]. Advantaged caste groups included the upper caste group (from both hilly and Terai region) and relatively advantaged Janajatis (Newar, Thakali, Gurung) [30]. Dis-advantaged caste group included Dalit (from both hilly and Terai region), dis-advantaged Janajati (hilly and Terai), religious minorities (Muslim) and dis-advantaged non-dalit Terai caste groups [30].

The final treatment outcome was dichotomised into ‘successful treatment outcome’ and ‘unfavorable treatment outcome’ variables [26]. Successful treatment outcome comprised patients classified as ‘cured’ and ‘completed treatment’ [26, 31], while unfavorable treatment outcome included ‘died’, ‘treatment failure’, ‘lost to follow up’ and ‘not evaluated’ [26, 27]. Participant age was categorised into groups according to weightage of the data i.e. ≤ 14, 15–24, 25–54 and ≥ 55 years, to facilitate comparison [29].

Data exploration involved descriptive and inferential statistics, following the process outlined in WHO toolkit ‘Incorporating intersectional gender analysis into research on infectious diseases of poverty: A toolkit for health researchers’ [15]. Sex-disaggregated data analysis was conducted in each step to identify difference between males and females across different ages and ethnic groups. Furthermore, we assessed whether any statistical difference existed between different age groups and ethnic groups within sex. Bivariate analysis employed Chi-square test to measure the association between available social stratifiers and types of TB and patient registration category. For variables with expected cell value less than five in bivariate analysis, the Fischer-exact test was applied. Multivariate logistic regression determined the most significant determinants associated with treatment outcome. Crude and adjusted odds ratios (*OR*s) were calculated during the analysis, with a 95% confidence interval (*CI*) used to report the *OR*.

## Results

### Where do we stand in terms of understanding inequalities in health system of Nepal? What is being done?

The Constitution of Nepal (2015) provides greater inclusion of female, marginalized and disadvantaged groups [18, 33]. Subsequently, there has been notable progress in biological and the social construct of gender approaches in various policies and strategies. These initiatives mandate civil society and economic participation, as well as health service utilisation by women. Gender, social inclusion and the concept of intersectionality are well incorporated into existing National Health Policy, Nepal Health Sector Strategy, Gender Equality and Social Inclusion Strategy of the Health Sector, Urban Health Policy, Population Policy and National Strategy for Reaching the Unreached [19–21, 23, 24].

In an endeavor to reach the unreached, the Ministry of Health and Population (MoHP) established a 'Gender Equality and Social Inclusion' (GESI) section in 2013. This proactive step aimed to address disparities and promote inclusivity by mainstreaming GESI in the health sector [34]. However, despite numerous efforts, the implementation of GESI policies faces challenges due to limited operational structures and capacity at various levels within the health system. Consequently, inequities in health outcomes persist across various social stratifiers [3, 35]. Challenges continue with the implementation of gender-sensitive and gender-responsive legislation, policies, and acts, including the intersectional recognition of factors affecting men or women based on ethnicity, caste, religion, language, indigeneity, marital status, occupation, geographical location, ability, and access to health and education [34, 36–38]. These interaction occur within connected systems where social determinants and the structure of power in the society synergistically and antagonistically act, forming the privilege and oppression of individuals [39].

Furthermore, in 2014/15, the MoHP revised the HMIS to include variables such as sex, age, caste/ethnicity, and location/address. This revision enables the assessment of disaggregated health data, offering a more comprehensive understanding of 11 selected health indicators [34, 40]. HMIS is primarily used in the public sector for recording and reporting routine health services data from public health facilities at all three levels of government (local, provincial, and federal). The private sector maintains its own information systems for recording purposes, which are not yet integrated with the government's HMIS. However, a few private health facilities report to HMIS for selected programme indicators only.

### Health Management Information System (HMIS): TB as a case example

HMIS in Nepal comprises distinct registers for recording TB service data, namely HIMS 6.1 Tuberculosis Sample Collection Form, 6.2 Tuberculosis Laboratory Register, 6.3 Tuberculosis Treatment Card (Health Facility), 6.4 Tuberculosis Treatment Card (Patient), 6.5 Tuberculosis Treatment Register, 6.6 Smoking cessation Register, 6.7 drug resistant (DR) Tuberculosis Laboratory Register, and 6.8 DR Tuberculosis Treatment Register [26]. All these TB registers typically include fields for recording demographic information, including age, sex, ethnicity, address, name of the caregivers and contact number of the service recipients. The classification of sex is limited to male and female, with no provision for individuals with non-binary gender identities.

**Fig. 1:**
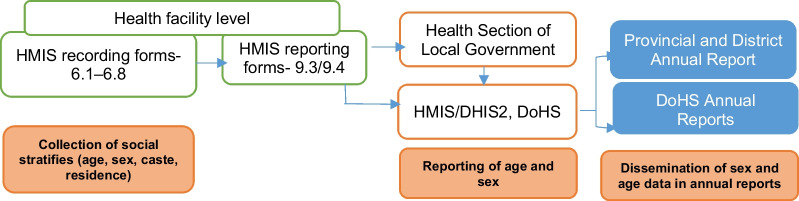
Flowchart presenting loss of variables during recording and reporting mechanism of TB. *DHIS2* District Health Information Software 2; *DoHS* Department of Health Services; *HMIS* Health management information system

While social stratifiers such as age, sex and ethnicity are recorded at the health facility levels, there are limitations in reporting this data to higher authorities. The standard reporting format predominantly focus on sex and age-disaggregated data. Information disseminated at the national level by the government through annual reports based on HMIS findings includes disaggregation by sex, age, and province. This highlights the gap, indicating that the health information system has limitations in understanding service utilisation patterns by different population groups to make tailored decisions and interventions (Fig. 1).

### Scope of conducting disaggregated and intersectional analysis from the available HMIS data: taking TB as an example

Secondary data analysis was performed to assess the current limitations in conducting intersectional gender analysis with the available TB data through the HMIS, rather than producing new findings to inform disease (TB) perspective. It is essential to note that the TB programme is taken only as an illustrative example. The insights gained from this analysis could contribute to inform HMIS recording and reporting practices for various diseases and health programmes, promoting a more inclusive system.

#### Trend of annually reported TB cases disaggregated by ecological region, age and sex

**Fig. 2:**
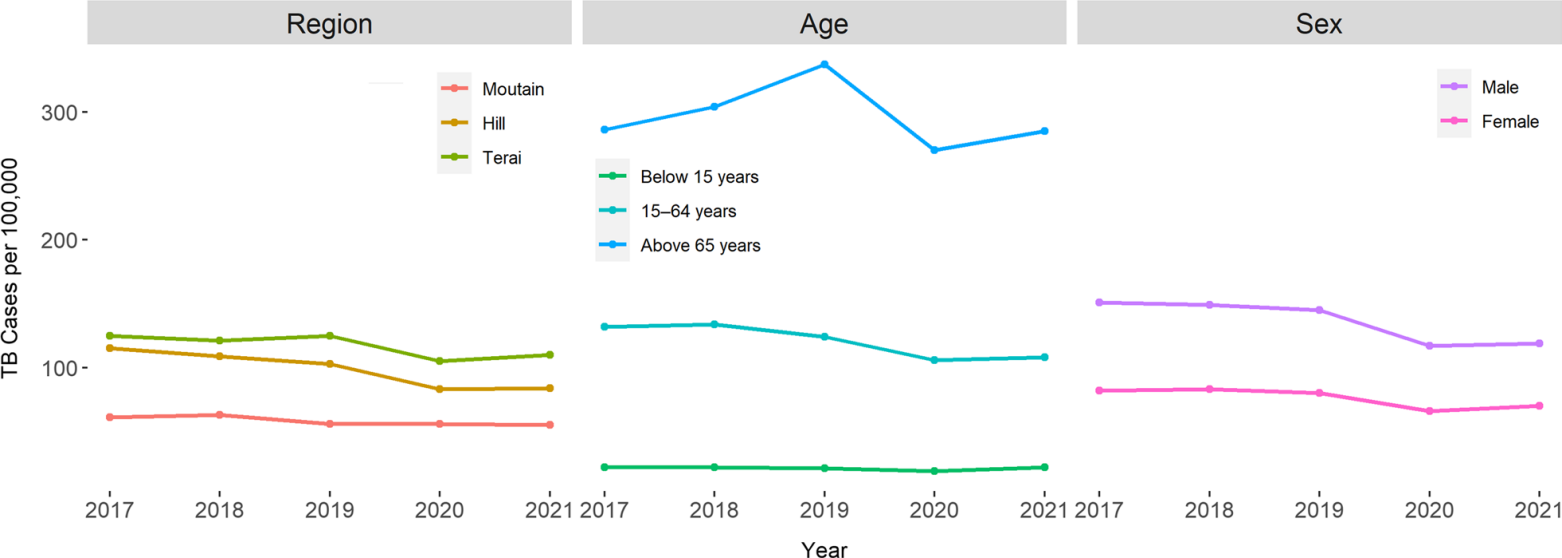
Tuberculosis cases by region, age, sex (Data Source-National Tuberculosis Control Center) [41]

There were pronounced variations in TB cases across different regions of Nepal. The Terai region (the lowland plains) consistently reported the highest TB cases, followed by the Hill region (the hilly areas) and the Mountain region (the mountainous areas) for the last five years. The highest proportion of TB cases was found among the population aged 65 years and above, whereas lowest proportion was found among less than 14 years. In terms of sex-wise distribution, the proportion of TB cases is notably higher among males compared to females over the last five years. These findings provide important insights into the epidemiology of TB in Nepal, showcasing variations in regional prevalence, age-related patterns, and gender disparities (Fig. 2).

#### Disaggregated analysis of the recorded TB cases

We collected information from 628 TB patients from two DOTS centers, among whom 510 (81.2%) were new TB patients, while 118 (18.8%) had received previous TB treatment. During the data collection period, 152 (24.2%) were under TB DOTS treatment and 476 (75.8%) had completed their treatment. Among the patients, 338 (54.0%) had pulmonary TB (PTB), and 290 (46.0%) had extra-pulmonary TB (EPTB). Of those who completed treatment, 399 (83.8%) were successfully treated, 71 (14.9%) had an unfavorable treatment outcome and 6 (1.3%) moved to second line treatment (data not shown).

The overall male-to-female TB patient ratio was 1.1 (333/295). The age distribution of male TB patients ranged widely from a minimum age of 9 months to a maximum age of 92 years. Similarly, the age diversity of female TB patients followed a similar pattern, ranging from a minimum age of one year to a maximum age of 93 years. However, median (md) and inter-quartile range (IQR) for the age of males (md = 34 years; IQR = 22–50) were higher than those of females (md = 27 years; IQR = 21–38). In both sexes, the highest percentage of TB patients belonged to the 25–54 years age group [male (46.6%) and female (45.4%)], while the ≤ 14 years age group had the lowest TB cases [male (5.7%) as well as female (4.4%)]. Similarly, more than half of the male (55.6%) and female (56.3%) TB patients belonged to advantaged caste group, while the remaining belonged to disadvantaged caste group (Table 1).

**Table 1:** Socio-demographic characteristics of the study participants

Characteristics	Male (*n* = 333, 53.0%) *n* (%)	Female (*n* = 295, 47.0%) *n* (%)	Total (*N* = 628) *n* (%)
Age (years)			
≤ 14	19 (5.7)	13 (4.4)	32 (5.1)
15–24	87 (26.1)	111 (37.6)	198 (31.5)
25–54	155 (46.6)	134 (45.4)	289 (46.0)
≥ 55	72 (21.6)	37 (12.5)	109 (17.4)
Median; Min; Max; IQR	34; 0.75; 92; 22–50	27; 1; 93; 21–38	30; 0.75; 93; 21– 45
Ethnicity			
Dis-advantaged caste group	148 (44.4)	129 (43.7)	277 (44.1)
Advantaged caste group	185 (55.6)	166 (56.3)	351 (55.9)

*Min* Minimum*, Max* Maximum

#### Comparison of types of TB according to age, sex and ethnicity

**Fig. 3:**
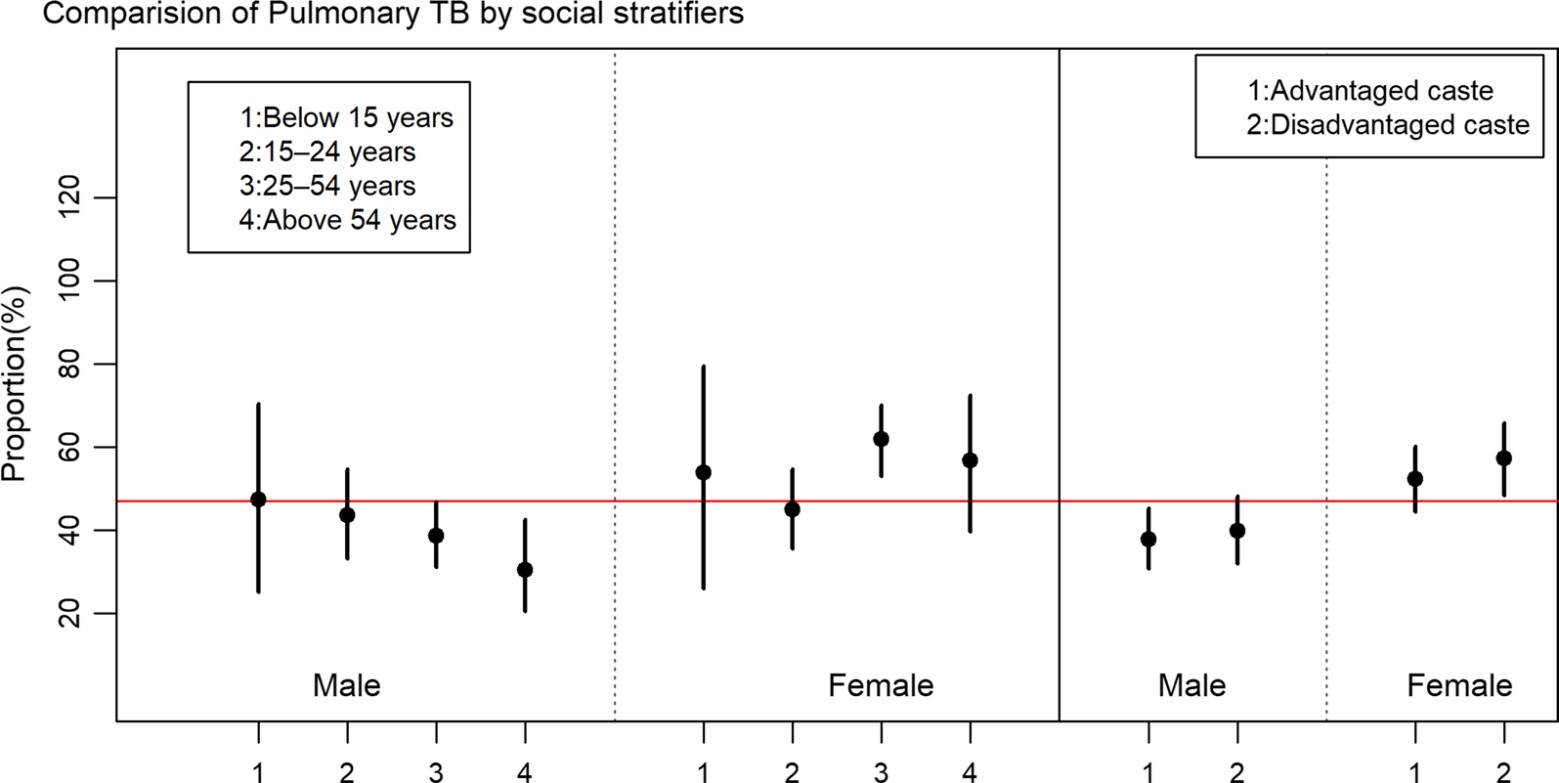
Comparison of Pulmonary TB cases by age and ethnic groups disaggregated by sex

There was a significant association between sex of the patient and the types of TB (*P* < 0.05). Among the reported cases, the proportion of males with PTB was higher (61.3%) compared to females (45.4%), while the proportion of males with EPTB was lower (38.7%) than that of females (54.6%). Figure 3 shows the proportion of pulmonary TB patients and their 95% confidence interval among different age and ethnic groups disaggregated by sex. The red horizontal line in Fig. 3 represents the proportion of pulmonary TB among total cases, i.e., 54.0%. Within males, the proportion of PTB increased with age, with the highest proportion of TB patients observed in the ≥ 55 years age group. Males had a higher prevalence of PTB compared to females in both, advantaged and dis-advantaged caste group (Fig. 3).

#### Patient’s registration category (old/new cases) across age, sex and ethnicity

Age and sex were significantly associated with patients’ types of TB cases during registration while enrolling into the TB regimen (*P* < 0.05). A significantly higher percentage of males (61.9%) sought retreatment compared to females (38.1%) (*P* < 0.05). Similarly, patients in the 25–54 years age group constituted a significantly higher proportion (44.1%) in the retreatment category. Although not statistically significant, a higher proportion (59.3%) of the disadvantaged caste group sought retreatment compared to the advantaged caste group (40.7%). While the difference is not statistically significant, it still underscores a noteworthy trend. (Table 2).

**Table 2:** Sex, age and ethnic groups by types of cases (old/new) during registration at DOTS center

Variables	Types of TB cases during registration			*χ*^2^(*df*)	*P*-value
	New (*n* = 510, 81.2%) *n* (%)	Previously treated patients (*n* = 118, 18.8%) *n* (%)	Total (*N* = 628) *n* (%)		
*Sex*				4.56(1)	0.033
Male	260 (51.0)	73 (61.9)	333 (53)		
Female	250 (49.0))	45 (38.1)	295 (47)		
*Age (in years)*				9.68 (3)	0.022
≤ 14	31 (6.1)	1 (0.8)	32 (5.1)		
15–24	162 (31.8)	36 (30.5)	198 (31.5)		
25–54	237 (46.5)	52 (44.1)	289 (46.0)		
≥ 55	80 (15.7)	29 (24.6)	109 (17.4)		
*Ethnic groups*				0.69(1)	0.405
Dis-advantaged	281 (55.1)	70 (59.3)	351 (55.9)		
Advantaged	229 (44.9)	48 (40.7)	277 (44.1)		

Fischer-exact test was applied in case of cell value less than 5; χ^2^-test was done

#### Treatment outcome across age, sex and ethnicity

**Fig. 4:**
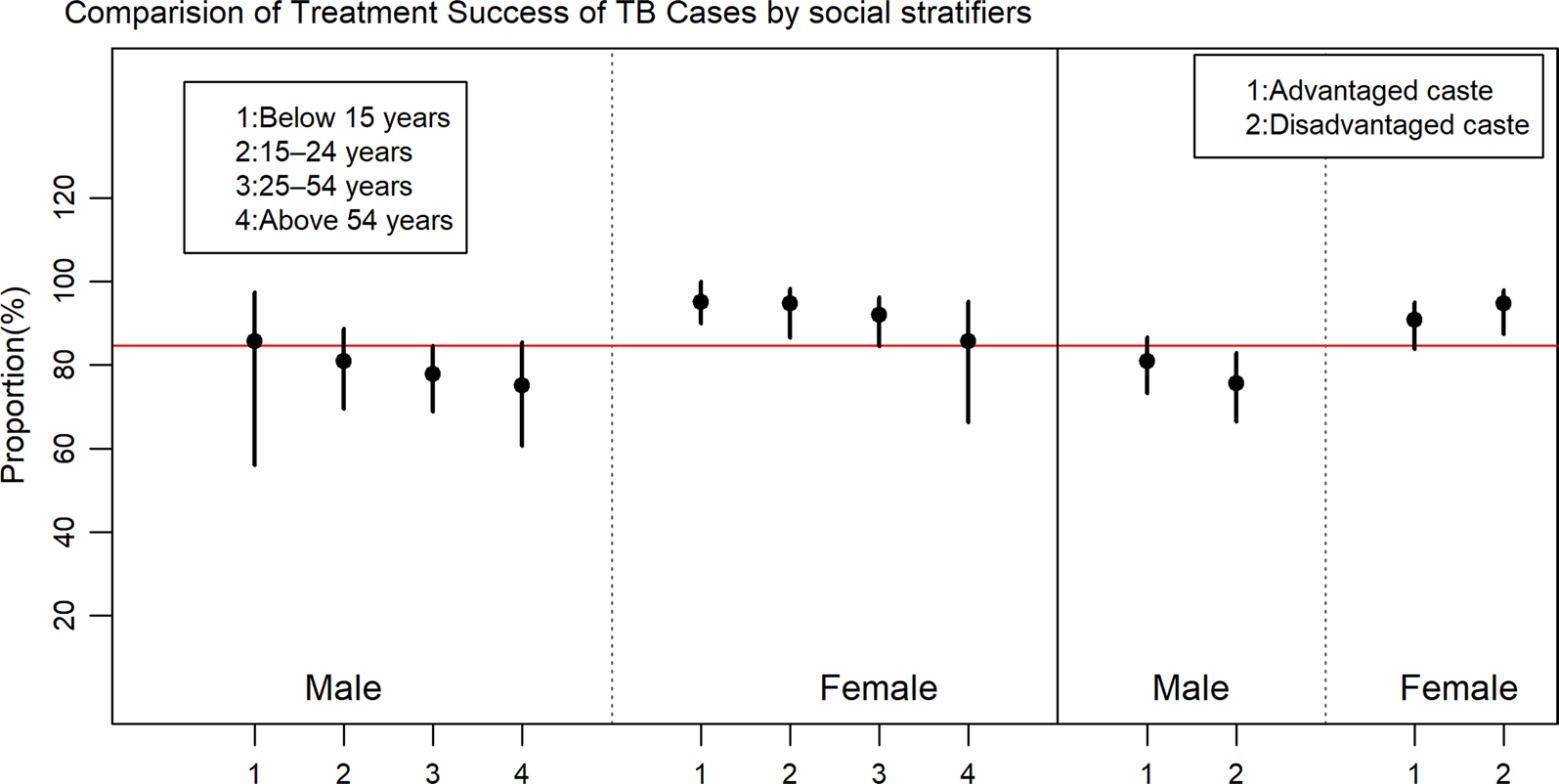
Comparison of treatment success rate of TB cases by age and ethnic groups disaggregated by sex

Out of 628 TB patients, a treatment outcome was obtained for 470 patients and 6 patients were moved to the second line treatment, which was not considered in the two categories of treatment outcome (successful and unfavorable) [32]. Figure 4 demonstrates the successful treatment outcome and its 95% confidence interval among age groups and ethnic groups disaggregated by sex. The red horizontal line represents the proportion of treatment success of TB patients among total TB patients i.e., 84.8%. The rate of successful treatment gradually decreased with age among both male and female TB patients. Female TB patients had higher successful treatment outcome in comparison to male across both caste groups.

Multivariate logistic analysis was conducted to assess the relationship between combined variables i.e. ‘sex and age’ and ‘sex and ethnicity’ and treatment outcome, where age group was categorized into two groups (≤ 25 years and > 25 years) due to insufficient sample size within four category of age groups. The results reveal that male more than 25 years exhibited higher odds (a*OR* = 4.95, 95% *CI*: 1.60–19.06, *P* = 0.01) of successful outcome compared to male TB patients less than 25 years (Table 3).

**Table 3:** Multivariate analysis between sex, age and ethnicity and treatment outcome

Variable	Treatment outcome					
	Unadjusted			Adjusted		
	*OR*	95% *CI*	*P-*value	*OR*	95% *CI*	*P*-value
*Sex and age*						
Male ≤ 25 years	1			1		
Male > 25 years	5.3	1.88–18.98	0.001	4.95	1.60–19.06	0.01
Female ≤ 25 years	0.85	0.45–1.59	0.62	0.83	0.43–1.56	0.57
Female > 25 years	2.3	1.06 –5.18	0.04	2.15	0.88–5.49	0.1
*Sex and ethnic groups*						
Male advantaged caste	1			1		
Male dis-advantaged caste	5.73	2.29–17.47	0.001	1.79	0.62–5.89	0.3
Female advantaged caste	1.36	0.75–2.48	0.31	1.38	0.75–2.51	0.3
Female dis-advantaged caste	3.19	1.54–7.03	0.001	2.56	0.91–4.55	0.09

0 = unfavorable outcome (treatment failure, died, loss to follow up, not evaluated); 1 = Successful treatment

## Discussion

Despite of numerous efforts to apply an intersectional gender lens in the policies, implementation has been mixed, leading to evident health inequities across various social stratifiers [3, 35]. The literature suggests that variations exist in the availability and utilization of health services, as well as health status of individuals based on several factors, including gender, age groups (with a special focus on vulnerable age groups), geography, urban/rural locations, socio-economic status, caste, ethnicity and religions, the presence of disabilities (both physical and mental) and disaster affected areas [38, 42, 43]. Moreover, multiple layers of vulnerability are created when two or more of these determinants intersect, amplifying the risks faced by excluded or marganilised populations. This shows the critical need for and importance of conducting gender and intersectional analysis in policy making and health planning, ensuring that no one is left behind and to addressing the specific needs of diverse and vulnerable population groups.

Although the importance of disaggregated data is emphasised in policies, its actual use for planning and developing programmes and interventions is limited. Another challenge lies in the fact that HMIS often have limited variables to record and report health service delivery data, restricting gender and intersectional analysis. This study highlights practical constraints in using existing HMIS for inclusive gender and intersectional analysis. However, it is unclear whether, how and what extent these information management systems in public and private sector provide gender and equity-focused evidence and how they inform decisions. All these challenges impede progress towards strengthening a health system that is more responsive and leaves no-one behind in federalized context. Therefore, more social stratifiers should be added to the HMIS recording and reporting forms, followed by incorporating intersectional gender lens while analysing and reporting the HMIS data. Such context highlights the complexity of addressing gender and social inclusion issues within the health sector in Nepal. While efforts have been made to recognise and tackle these challenges, practical implementation remains a significant hurdle due to capacity gaps, resource constraints and the limitations for comprehensive data collection and analysis. Addressing these challenges is crucial for achieving more equitable health outcomes across diverse social groups.

Intersectional analysis of HMIS recorded data conducted in this study illustrated various differences across sex, age and ethnicity. The proportion of females with EPTB was higher than males, consistent with studies in the United Denmark [16], and India [17]. Several factors, such as endocrine factors, smoking, and past history of TB exposure were thought to be related to this inequality [36, 38].

Sex was significantly associated with the treatment success rate where a greater proportion of females had favorable treatment compared to males. Analysis of gender differentials has indicated that women who begin treatment for TB are more likely to adhere to the full course of treatment compared to men, resulting in a positive treatment outcome [40, 41]. Men, being sole breadwinners, are engaged in various informal sectors and have less chance to become aware of the disease; hence, the probability of treatment non-adherence is high [32]. This continues as a cycle of TB, where a high proportion of male TB patients came for re-treatment of TB compared to female, as evidenced in this study. Furthermore, this study identified that the treatment success rate gradually decreases with an increase in age among both sexes, aligning with other studies [45, 46]. This could be because older TB patients interrupt adherence to treatment more often than younger persons and are challenged by several determinants of health, such as low socioeconomic status, low immunity and poor access to health facility [47]. Therefore, older persons with TB might benefit from close monitoring in order to make their treatment successful [48].

Our study did not identify significant differences in TB-related outcomes across ethnic groups. However, various studies conducted in other countries have shown that the migrant population and ethnic minorities have a higher prevalence of TB in comparison to the general population [49–53]. This could be because of interactions between cultural and structural barriers to accessing healthcare [3, 4, 50–53]. Behind this, social power and structures have influenced vulnerability and treatment outcome of TB among people living in slums and densely populated urban settings, people living in congregate settings like factories, prisons, camps and refugees [44]. With limitations on disaggregated population data in the routine healthcare information system and a lack of context-specific models for identification and determining numbers and distribution of high-risk groups, there is less effective coverage of priority health interventions among these groups[14]. This has resulted in difficulties in the timely diagnosis of TB and prompt initiation of treatment [14].

Apart from these, other studies shows that, even though anti-TB medicines are provided free of cost, various factors such as socio-economic conditions, fear of losing job, lack of education, ethnicity as a cross cutting factor, family responsibilities contribute to the loss to follow up during TB treatment [16, 17]. Because of these reasons, sex, gender, age, education, occupation, race/ethnicity and residential area of TB patients also interplay with each other to influence the treatment outcome of TB [16, 17]. Hence, if we could move towards specific approaches of recording, reporting, and analysing of TB cases according to social strata (age, sex, ethnicity, education, occupation, province, etc.) of TB patients, this would contribute to narrowing down the existing information gap and identifying the unreached population.

There are some limitations in our study. Secondary data was used for the study which limited the scope of variables of this study as social stratifiers recorded in the HMIS 6.5 register of the TB was just confined to age, sex and ethnicity of the patient. This narrowed down the opportunity to conduct intersectional gender analysis to the wider extent. Also, treatment outcome of all the TB patients from the collected data could not be analysed across social stratifiers because 152 patients were still under TB treatment regimen during the time of data collection, for which treatment outcome was awaited. This ultimately reduced our sample size while analyzing ‘treatment outcome’ for this study.

## Conclusions

The intersectional analysis conducted with limited variables (age, sex and ethnicity) presented differences across treatment outcome and types of TB within different age group and ethnicity of male and female TB patients. Hence, this study reflected the potential of reaching the unreached or vulnerable group of population via intersectional gender analysis when range of social stratifiers are captured, analysed and evidence-based decision is taken. Similarly, the findings highlight the inadequacy in the availability of social stratifiers in routine HMIS TB data. This limitation hampers the NTP’s ability to conduct intersectional analysis, essential for unveiling the roles and impacts of various social determinants of TB. Such limitation underscores the necessity for more disaggregated and inclusive data in routine NTP HMIS, enhancing the ability to inform policies and plans for building a more responsive and equitable TB programme that can systematically address disparities in TB outcomes.

## Data Availability

The datasets generated during this study are not publicly available due data confidentiality policy but are available from the corresponding author on reasonable request.

## Abbreviations

a*OR*: Adjusted odds ratio
*CI*: Confidence interval
DHIS2: District Health Information Software 2
DoHS: Department of Health Services
DR: Drug resistant
DOTS: Directly observed therapy, short-course
EDCD: Epidemiology and Disease Control Division
EPTB: Extra-pulmonary tuberculosis
GESI: Gender Equality and Social Inclusion
GENETUP: German Nepal Tuberculosis Project
HMIS: Health Management Information System
IQR: Inter-quartile range
MOHP: Ministry of Health and Population
NATA: Nepal Anti-Tuberculosis Association
NTCC: National Tuberculosis Control Center
NTP: National Tuberculosis Programme
OR: Odds ratio
PTB: Pulmonary tuberculosis
TB: Tuberculosis
UHC: Urban Health Clinics
WHO: World Health Organization
